# Quality Prediction for Injection Molding by Using a Multilayer Perceptron Neural Network

**DOI:** 10.3390/polym12081812

**Published:** 2020-08-12

**Authors:** Kun-Cheng Ke, Ming-Shyan Huang

**Affiliations:** Department of Mechatronics Engineering, National Kaohsiung University of Science and Technology, 1 University Road, Yanchao Dist., Kaohsiung City 824, Taiwan; kcke@nkust.edu.tw

**Keywords:** cavity pressure, injection molding, intelligent manufacturing, multilayer perceptron model, quality prediction

## Abstract

Injection molding has been widely used in the mass production of high-precision products. The finished products obtained through injection molding must have a high quality. Machine parameters do not accurately reflect the molding conditions of the polymer melt; thus, the use of machine parameters leads to erroneous quality judgments. Moreover, the cost of mass inspections of finished products has led to strict restrictions on comprehensive quality testing. Therefore, an automatic quality inspection that provides effective and accurate quality judgment for each injection-molded part is required. This study proposes a multilayer perceptron (MLP) neural network model combined with quality indices for performing fast and automatic prediction of the geometry of finished products. The pressure curves detected by the in-mold pressure sensor, which reflect the flow state of the melt, changes in various indicators and molding quality, were considered in this study. Furthermore, the quality indices extracted from pressure curves with a strong correlation with the part quality were input into the MLP model for learning and prediction. The results indicate that the training and testing of the first-stage holding pressure index, pressure integral index, residual pressure drop index and peak pressure index with respect to the geometric widths were accurate (accuracy rate exceeded 92%), which demonstrates the feasibility of the proposed method.

**Highlights**

1. A single-hidden-layer multilayer perceptron (MLP) neural network inspection system was developed to predict the geometric quality of molding parts.
2. This study refers to the pressure profiles of the polymer melt, which reflect the flow behavior in the cavity during the injection molding process, rather than considering traditional machine parameters of injection molding machines.
3. Eleven quality indices were extracted from the pressure profile and the indices having high correlation coefficients with the geometric width were used as the input data of the MLP model.
4. The optimal training accuracy of the MLP model for predicting the geometric quality exceeded 93%. Moreover, the prediction accuracy of the MLP model was more than 92% for three geometric widths.

## 1. Introduction

**Figure 1:**
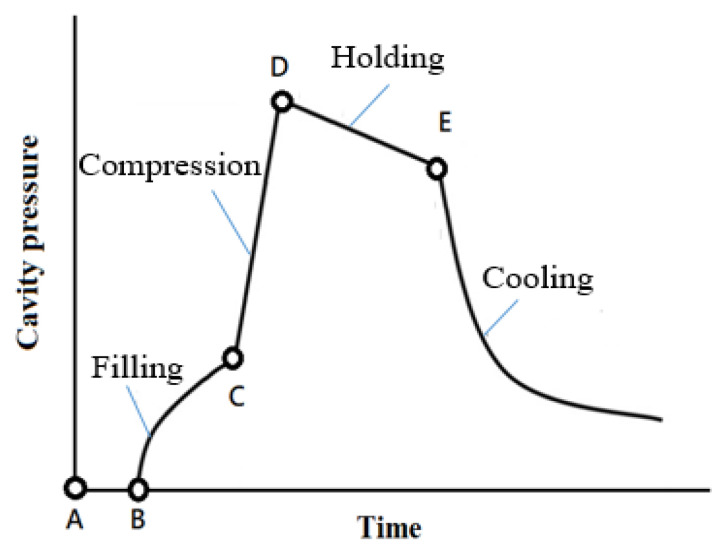
Typical cavity pressure profile.

Injection molding has been widely used in the large-scale manufacturing of high-precision products, which involves four main phases—filling, compression, holding and cooling. The aforementioned manufacturing process is considered a black-box process because the flow behavior of the polymer melt in the mold cavity is not visible. Traditional quality control based on the machine parameters of the injection molding machine has limitations, which lead to incorrect judgments of the part quality [1]. With the advancement of sensing technology, many sensors, such as pressure sensors, can be used to understand the flow behavior of the polymer melt in the mold cavity. Cavity pressure has been proven to determine the repeatability of the injection molding quality [2]. Figure 1 displays a typical cavity pressure curve, where the filling process begins at point A and the cavity pressure signal begins at point B. The polymer melt initially contacts the pressure sensor. The pressure then increases steadily as the filling process progresses. The filling phase ends at point C, at which the cavity is volumetrically filled with the polymer melt without being compressed. The compression process then begins and the pressure quickly rises to a peak at point D. Therefore, during the holding phase, as additional polymer melt enters the mold cavity to compensate for plastic shrinkage, the melt in the cavity is maintained at a specific pressure. This process continues until the point of the gate (indicated by point E) is sealed. Subsequently, the final cooling phase occurs and continues until the end of the cycle. In this phase, as coolant circulation in the cooling channels in the mold results in a decrease in heat, the polymer melt gradually solidifies. The cooling and solidification rates determine the downward trend of the cavity pressure.

Polymer materials used in injection molding are sensitive to temperature changes. Shrinkage and warpage of molded parts that often occur in injection molding can be attributed to part geometry, material properties and processing parameter settings. Without concerning the influence of part geometry, the quality of molded parts is affected by controllable and uncontrollable factors. The controllable factors are the process parameter settings, especially the melt and mold temperatures, injection speed and pressure, velocity-to-pressure (V/P) switchover and holding pressure and time. The uncontrollable factors are related to material variation between batches and environmental changes. If the aforementioned two types of factors are maintained in a stable state, consistent part quality can be ensured and production costs can be reduced [3]. Therefore, online measurement of the polymer melt flowing ability is critical for monitoring process conditions [4]. For instance, Cornik [5] developed a device mounted on the nozzle of an injection molding machine to measure online the rheological property of the polymer melt. In other words, the melt flow index was used as a quality index for each batch of materials. Aho et al. [6] used the ratio between the pressure gradient and volumetric flow rate to calculate the viscosity. Ogorodnyk and Martinsen [7] also mounted pressure sensors on the nozzle of an injection molding machine to measure the polymer melt quality. In addition, by combining the apparent viscosity of the melt, which is calculated using a pressure sensor, with the melt temperature, an index indicating the quality of the melt can be obtained. Similarly, techniques have been developed to detect the melt pressure, temperature and viscosity by using multiple sensors for determining the melt quality [8,9]. Another method of monitoring the molding conditions is to observe the tie bar elongation at each shot, which is not invasive to the mold structure. Chen et al. [10] suggested that by checking the elongation signals of the tie bar, appropriate values of the clamping force can be determined, which can enhance the molding quality, reduce energy consumption and increase the mold life. Moreover, by changing the V/P switchover, the quality of injection molding can be improved with each injection [11].

At present, many studies mention how to use sensor technology to convert the performance of polymer melts into quality indexes and then apply them to actual quality prediction and control. For example, Chen et al. [12] explored the relationship between injection process parameters and part quality and revealed that injection molding process control can be divided into four levels—process condition setting, machine control, process control and quality control. Farahani et al. [13] used in-mold sensors for quality monitoring, of which the partial least square method is used to establish a mathematical model of quality indexes and part quality.

Statistical methods are often used to evaluate the factors affecting the quality of injection molding. For instance, Zhang et al. [14] used principal component analysis and analysis of variance to analyze the key factors affecting the injection molding quality statistically. In their research, the warpage of the molded parts was appropriately controlled using statistical tools and data mining techniques for manipulating the cooling parameters. Zhang et al. observed that the flow rate of coolant channels considerably affected the warpage of the molded parts. Moreover, they established a fourth-order ARX model to describe the relationship between the part weight and the mold temperature. This model can be used as a weight estimator.

With the trend of intelligent manufacturing, the accuracy and automation of injection molding can further be improved through artificial intelligence (AI) [15], cyber-physical systems [16], Internet of Things [17] and data mining [18]. AI is a method that combines domain, statistical and computer science knowledge by simulating human intelligence. Yeh et al. [19] used a decision tree algorithm to establish an intelligent molding test classification knowledge system. The prediction accuracy of the developed model was approximately 87%. Raviwongse et al. [20] developed an efficacious design tool by using a backpropagation neural network (BPNN). The tool can perform complex mold design, including part geometry, parting line, material and cavity design. Ogorodnyk et al. [21] used multilayer perceptron (MLP) models and decision trees to predict the tensile strength of high-density polyethylene samples. Shen et al. [22] combined the BPNN and genetic algorithms to optimize injection molding parameters. In addition, Bensingh et al. [23] integrated the hybrid artificial neural network and particle swarm optimization methods to optimize the process parameters for fabricating a bi-aspheric lens. A good agreement was observed between the predicted and actual curvature of the bi-aspheric lens. The difference in the predicted and actual curvature was less than 1%. Machine learning (ML) and deep learning (DL) can be used to build quality prediction models can employ, which can effectively non-linear fit input and output data. Currently, conducting ML and DL by using open source code and modules, such as Matlab [24] and Python [25], for programming is not only efficient but also cost-effective. In addition, cloud computing platforms, such as Amazon, Azure and Google Colab, provide complex hardware and various training modules for ML, which allow users to perform remote operations and reduce hardware costs [26,27,28,29].

In summary, many scholars have used artificial intelligence technology to predict the quality of injection parts and achieved the effect of intelligence and automation. However, the input or learning information used is often a mechanical setting parameter. In this way, not only can it not accurately respond to the response problems of different injection machines of the same factory but also it is impossible to accurately grasp the product quality changes caused by the melting glue variation during the injection process. Therefore, establishing product quality in a scientific way is a solution that is urgently needed. According to the cavity pressure, which indicates the flow behavior of the polymer melt in the mold cavity and the quality of the molded part, this study developed a quality prediction system for predicting the geometric width of molded parts by using various quality indices extracted from the in-mold pressure profile and MLP models based on the Google Colab platform.

## 2. Methodology

### 2.1. Experiment Design

Machine parameters, such as the injection speed and holding pressure, are typically selected as independent variables. In the performed experiment, the injection speed and first-stage holding pressure were selected as independent variables. A two-factor full-factor experiment was used to obtain quality data affected by the two selected variables. The width deviation of the final product was selected as the dependent variable. The injection speed and the holding pressure in the first stage influence the width deviation of the molded part. However, the quality of the injection-molded part depends on the flow characteristics of the polymer melt in the mold cavity, especially the cavity pressure, rather than the machine parameters. Therefore, this study used the cavity pressure information of the polymer melt in the cavity to predict the quality of the final product. Moreover, various physical indices closely related to the quality of injection molding were used to train the quality prediction model.

The width deviation is a function of the cavity pressure, can be obtained from the in-mold sensor setting and is affected by the machine parameters injection speed and holding pressure in the first stage. The cavity pressure profile displays the flowing course of the polymer melt in the cavity during injection molding (Figure 1). An examination of the pressure signal in the time domain indicates that the melt property changes at each stage in the injection process. Furthermore, the features of the cavity pressure profile can be extracted to design quality indices that represent the quality of the final product.

This study also used Pearson’s correlation coefficient (PCC) to verify the correlation between quality indices and quality. Equation (1) presents the equation for PCC (*r*), whose value is between −1 and 1. Table 1 lists the correlation strength (related to *r*). The higher the value of *r*, the stronger is the correlation between two variables. Thus, variables with high *r* values trend in the same direction. In this study, an *r* value greater than 0.75 related to the quality of injection molding was selected as the independent variable and used in the input layer of the machine learning model:

(1) $$r=\frac{\sum^{\;}x_{i}y_{i}-n\bar{x}\bar{y}}{\sqrt{\left(\sum^{\;}x_{i}{}^{2}-n{\bar{x}}^{2}\right)\left(\sum^{\;}y_{i}{}^{2}-n{\bar{y}}^{2}\right)}}$$

### 2.2. Quality Indices

To predict the part quality corresponding to changes in the molding conditions, this study evaluated various quality indices. These indices, which were highly correlated with the part quality, were used instead of process parameters as input parameters for model training, which enhanced the prediction accuracy. By referring to the pressure signals, we selected the following indices as quality indices:

1. First-stage holding pressure index $\left(Ph_{index}\right)—$ in Equation (2), $Ph_{index}$ represents the average holding pressure in the first stage and $t_{0}$ and $t_{1}$ represent the beginning and end of the holding in the first stage, respectively. The holding process, which is also called post-filling, involves compensating for the cavity gap caused by the shrinkage of the polymer melt. This process is critical to the geometric quality of the molding part.
  (2) $$Ph_{index}=\frac{1}{t_{1}-t_{0}}\int_{t_{0}}^{t_{1}}g\;dt$$
2. Peak pressure index ($Pp_{index}$)—in Equation (3), $Pp_{index}$ represents the maximum pressure during the filling and compression process. In the injection molding process, the role of pressure is to drive the polymer melt to fill the cavity. The maximum pressure affects the amount of polymer melt filled into the cavity, which determines the geometric quality of the injection-molded part.
  (3) $$Pp_{index}=\mathrm{Max}\left(g\right)$$
3. Residual pressure drop index ($Pr_{index}$)—in Equation (4), $Pr_{index}$ represents the average residual cavity pressure drop during the cooling process; $t_{2}$ represents the end of holding, that is, the beginning of cooling; and $t_{3}$ represents the end of cooling. The average residual pressure drop is related to the residual stress in the processed polymer. High average residual pressure may cause geometric warping and low average residual pressure may cause undersizing of the molded part.
  (4) $$Pr_{index}=\frac{1}{t_{3}-t_{2}}\int_{t_{2}}^{t_{3}}g\;dt$$
4. Pressure integral index ($PI_{index}$)—in Equation (5), $PI_{index}$ is the integral of the pressure curve with time in a molding cycle (i.e., from filling to compression, holding and finally, cooling). This index is related to the overall pressure characteristics of the polymer melt during the injection molding process. Deviations may reflect changes in part quality, particularly weight changes [30].
  (5) $$PI_{index}=\int_{0}^{t_{3}}g\;dt.$$

The aforementioned four indices with different numerical levels are normalized using Equation (6). When these indices are used as input data for model training, fast convergence and accuracy can be achieved. In Equation (6), $X_{norm,\;i}$, $X_{max}$*,* $X_{min}$ and *N* represent the normalized value of $X_{i}$, the maximum value of *X*, the minimum value of *X* and the number of data, respectively. The range of $X_{norm,i}$ is set between 0 and 1.

(6) $$X_{norm,i}=\frac{X_{i}-X_{min}}{X_{max}-X_{min}}$$

### 2.3. MLP Model

**Figure 2:**
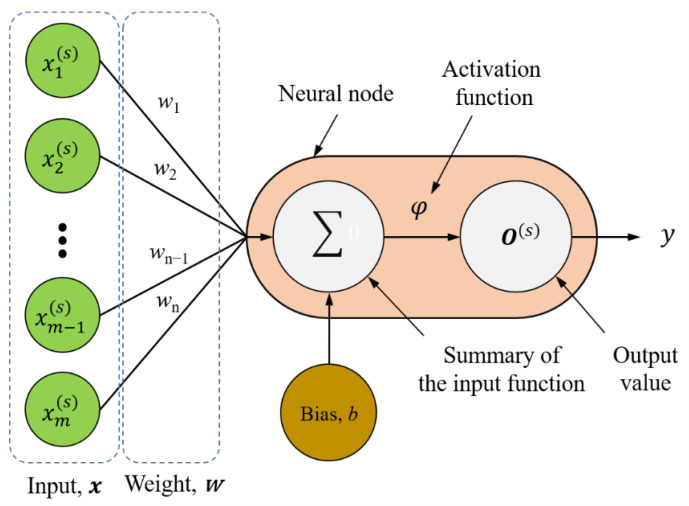
Single-neuron perceptron model.

A supervised artificial neural network learning model, which typically consists of three main parts, namely an input layer, hidden layers and an output layer, was used as the MLP model in this study [31]. The input layer receives input vectors and then passes each input data point to the neurons in the hidden layer. Neurons (also called neural nodes) in the hidden layer contain a summation function and an activation function. Figure 2 illustrates a single-neuron perceptron model, in which the activation function φ (Equation (7)) is a nonlinear function used to map the summation function (***xw*** + b) to the output value *y*. The terms ***x***, ***w***, *b* and *y* represent the input vector, weighting vector, bias and output value, respectively.

(7) y=φ(xw+b)

**Figure 3:**
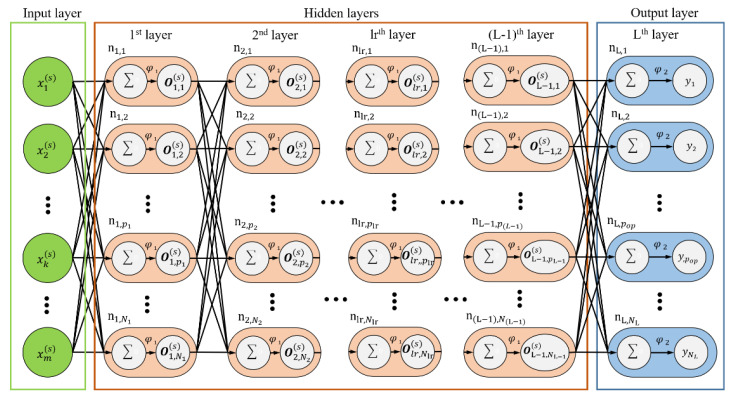
Structure of the multilayer perceptron (MLP) model.

Figure 3 illustrates the structure of the MLP model. In the figure, $x_{k}^{\left(s\right)}$ represents the *k*th input data at the *s*th set of data, *m* represents the total number of input data, $n_{lr,p_{lr}}$ represents the $p_{lr}$th neural node of the lrth layer and *N_lr_* represents the total number of neurons in the lrth layer. The notation $x^{\left(s\right)}$ represents the vector of input data; *N_set_* represents the total number of data points in the input dataset; and *L* represents the summation of the layers except the input layer. Equations (8)–(10) present the expressions for the output vectors of the first layer, *lr*th layer in the hidden layer and output layer, respectively. In the aforementioned equations, ***w****_lr_* represents the weighting vector of the *lr*th layer. The weighting values range between 0 and 1. These values change with the training data and represent the memory of the neural network related to the input and output after model training.

For lr=1,

(8) $$O_{1}^{\left(s\right)}=\varphi_{1}(x^{\left(s\right)}\times w_{1}+b_{1}{}^{T})$$

where

(8a) $$O_{1}^{\left(s\right)}=\left[\begin{array}{ccc} O_{1,1}^{\left(s\right)} & O_{1,2}^{\left(s\right)} & \begin{array}{cc} \cdots & O_{1,N_{1}}^{\left(s\right)} \end{array} \end{array}\right]$$

(8b) $$w_{1}=\left[\begin{array}{c} \;w_{1,1,1}\;\;\\ w_{1,1,2}\;\;\\ \begin{array}{c} \begin{array}{c} \vdots \\ w_{1,1,m} \end{array} \end{array}\;\; \end{array}\begin{array}{c} w_{1,2,1}\;\\ w_{1,2,2}\;\\ \begin{array}{c} \begin{array}{c} \vdots \\ \;w_{1,2,m} \end{array} \end{array}\; \end{array}\begin{array}{c} \;\cdots \;\\ \begin{array}{c} \ddots \\ \begin{array}{c} \cdots \end{array} \end{array}\;\; \end{array}\begin{array}{c} \;w_{1,N_{1},1}\;\;\\ w_{1,N_{1},2}\;\;\\ \begin{array}{c} \begin{array}{c} \vdots \\ w_{1,N_{1},m} \end{array} \end{array}\;\; \end{array}\right]$$

(8c) $$b_{1}=\left[\begin{array}{c} b_{1,1}\\ b_{1,2}\\ \begin{array}{c} \vdots \\ b_{1,N_{1}} \end{array} \end{array}\right]$$

For *L* > *lr* ≥ 2,

(9) $$O_{lr}^{\left(s\right)}=\varphi_{1}\left(O_{lr-1}^{\left(s\right)}\times w_{lr}+b_{lr}{}^{T}\right)$$

where

(9a) $$O_{lr}^{\left(s\right)}=\left[\begin{array}{ccc} O_{lr,1}^{\left(s\right)} & O_{lr,2}^{\left(s\right)} & \begin{array}{cc} \cdots & O_{lr,N_{lr}}^{\left(s\right)} \end{array} \end{array}\right]$$

(9b) $$w_{lr}=\left[\begin{array}{c} w_{lr,1,1}\;\;\\ w_{lr,1,2}\;\;\\ \begin{array}{c} \begin{array}{c} \vdots \\ \;w_{lr,1,N_{lr-1}} \end{array} \end{array}\;\; \end{array}\begin{array}{c} w_{lr,2,1}\;\\ w_{lr,2,2}\;\\ \begin{array}{c} \begin{array}{c} \vdots \\ w_{lr,2,N_{lr-1}} \end{array} \end{array}\; \end{array}\begin{array}{c} \;\cdots \;\\ \begin{array}{c} \ddots \\ \begin{array}{c} \cdots \end{array} \end{array}\;\; \end{array}\begin{array}{c} w_{lr,N_{lr},1}\;\;\\ w_{lr,N_{lr},2}\;\;\\ \begin{array}{c} \begin{array}{c} \vdots \\ w_{lr,N_{lr},N_{lr-1}} \end{array} \end{array}\;\; \end{array}\right]$$

(9c) $$b_{lr}=\left[\begin{array}{c} b_{lr,1}\\ b_{lr,2}\\ \begin{array}{c} \vdots \\ b_{lr,N_{lr}} \end{array} \end{array}\right]$$

For lr=L,

(10) $$O_{L}^{\left(s\right)}=\varphi_{2}\left(O_{L-1}^{\left(s\right)}\times w_{L}+b_{L}{}^{T}\right)$$

where

(10a) $$O_{L}^{\left(s\right)}=\left[\begin{array}{ccc} O_{lr,1}^{\left(s\right)} & O_{lr,2}^{\left(s\right)} & \begin{array}{cc} \cdots & O_{lr,N_{lr}}^{\left(s\right)} \end{array} \end{array}\right]$$

(10b) $$w_{L}=\left[\begin{array}{c} w_{L,1,1}\;\;\\ w_{L,1,2}\;\;\\ \begin{array}{c} \begin{array}{c} \vdots \\ \;w_{L,1,N_{L-1}} \end{array} \end{array}\;\; \end{array}\begin{array}{c} w_{L,2,1}\;\\ w_{L,2,2}\;\\ \begin{array}{c} \begin{array}{c} \vdots \\ w_{L,2,N_{L-1}} \end{array} \end{array}\; \end{array}\begin{array}{c} \;\cdots \;\\ \begin{array}{c} \ddots \\ \begin{array}{c} \cdots \end{array} \end{array}\;\; \end{array}\begin{array}{c} w_{L,N_{L},1}\;\;\\ w_{L,N_{L},2}\;\;\\ \begin{array}{c} \begin{array}{c} \vdots \\ w_{L,N_{L},N_{L-1}} \end{array} \end{array}\;\; \end{array}\right]$$

(10c) $$b_{lr}=\left[\begin{array}{c} b_{L,1}\\ b_{L,2}\\ \begin{array}{c} \vdots \\ b_{L,N_{L}} \end{array} \end{array}\right]$$

The term $O_{lr}^{\left(s\right)}$ represents the output vector of the *lr*th layer after training from the first dataset to the *s*th dataset. With regard to the activation functions used in this study, Equations (11) and (12a,b) present the expressions of the sigmoid function used in the hidden layers and the softmax function used in the output layer, respectively. In binary classification, the sigmoid function (also known as a logical function) maps the summary of the input function to the interval (0, 1). The softmax function, which is also known as the softargmax or normalized exponential function, is a function that normalizes an input vector of $N_{L}$ real numbers into a probability distribution consisting of $N_{L}$ probabilities proportional to the exponentials of the input numbers. After applying the softmax function, the component is in the interval (0, 1) and its summary is 1. Large input components correspond to large probabilities. Thus, the softmax function maps the non-normalized output of a network to a probability distribution over predicted output classes.

(11) $$\varphi_{1}\left(o_{i}\right)=\frac{1}{1+e^{-o_{i}}}$$

(12a) $$\varphi_{2}\left(o_{i}\right)=\frac{e^{o_{i}}}{\sum_{j=1}^{N_{L}}e^{o_{j}}}$$

(12b) $$\underset{i}{\sum }\varphi_{2}\left(o_{i}\right)=1$$

This study used the accuracy function *A_i_* presented in Equation (13) to evaluate the convergence of model training. The training accuracy can be evaluated by comparing the predicted value and actual output value. For instance, if the predicted value is consistent with the actual value, the weighting value is recorded and training is continued for the next dataset; otherwise, the weighting value is adjusted. The terms $N_{mis,i}$, $N_{total}$ and $N_{train}$ in Equation (13) represent the number of misclassified data points at the *i*th training iteration, the total number of data points and the total number of training iterations, respectively. As the number of training iterations increases, the distribution of the accuracy function converges to a constant value. By setting the stopping criteria, we can obtain high-quality results in model training.

(13) $$A_{i}=\left(1-\frac{N_{mis,\;i}}{N_{total}}\right)\times 100\%$$

The number of hidden layers and neurons affects convergence. MLP models with a large number of neurons and layers require numerous calculations for the weighting values, which may cause divergence in the predicted and actual values. MLP models with few neurons or layers may not generate a good connection between the input and output layers; thus, the predicted values of these models may be unstable. Moreover, data errors from experiments may lead to incorrect results in model training. Thus, a precise injection molding machine and precise measuring equipment must be used to ensure the prediction quality of the trained model.

### 2.4. Width Measurement and Quality Classification

**Figure 4:**
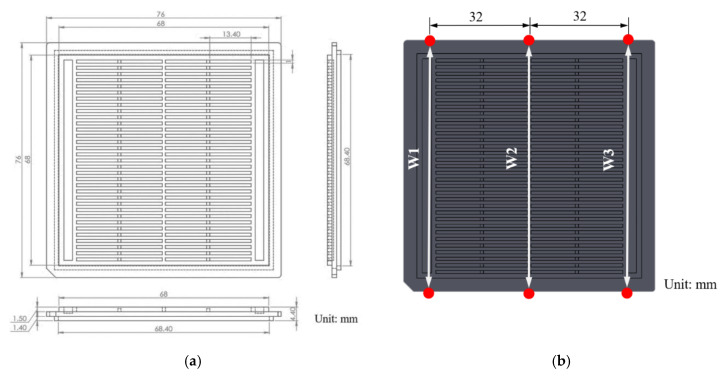
(**a**) Geometry of the Integrated Circuit (IC) tray and (**b**) six measurement positions for width deviations.

**Figure 5:**
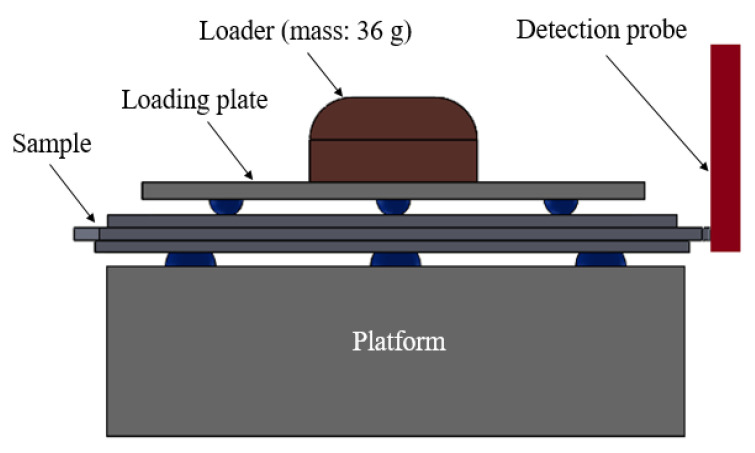
Steps in sample measurement: (a) placing the sample and IC tray on the jig; (b) applying a 36-g loading force to fix sample and (c) measuring the width with the probe.

Figure 4 illustrates the geometry of the Integrated Circuit (IC) tray manufactured in this study. The three geometric widths of the manufactured part were considered as critical qualities. The three widths (*W1*, *W2* and *W3*) were measured using a precise coordinate measuring machine (CRYSTA-Apex S700, Mitutoyo Corporation, Kawasaki, Japan). A laboratory-made fixture (Figure 5) was used to hold the sample under test so that when the detection probe touched the edge of the sample, the movement of the IC tray was minimized. Initially, the IC tray was fixed between the loading plate and the platform, with three hemispheres on the loading plate and a 36-g loader on the other side for stabilizing the sample on the platform. The detection probe used in the measurement was a cylinder with a diameter of 4 mm. The probe touched several points at the edge of the sample to measure the width. The coordinates of the measurement data were then converted to obtain the geometric widths.

**Figure 6:**
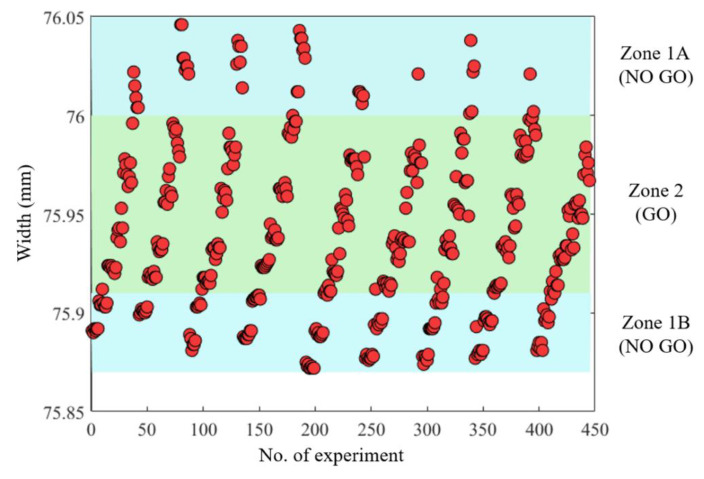
Classification of *W1*.

The width ranges were divided into three zones, as displayed in Figure 6. Zone 2 represented widths of good quality (Go) and Zones 1A and 1B represented widths of poor quality (No Go). Oversized and undersized parts were classified into Zones 1A and 1B, respectively. The width values were quantified to three decimal points to increase the stability of model learning.

## 3. Experimental

**Figure 7:**
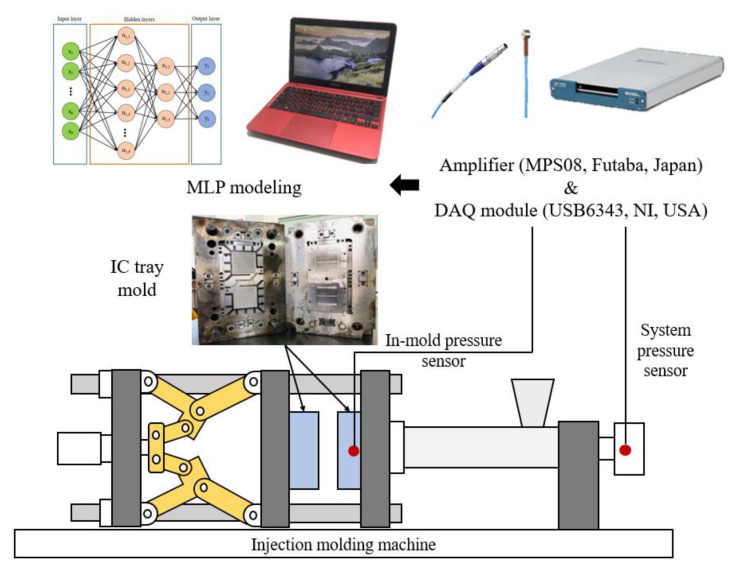
Schematic of the MLP inspection system.

Figure 7 illustrates a schematic of the designed MLP inspection system, including an injection molding machine, an injection mold for manufacturing IC trays, two types of pressure sensors, a data acquisition module and a computer for MLP modeling. The details of each component are provided in the following sections.

### 3.1. Injection Machine, Material, Mold and Sensor

**Figure 8:**
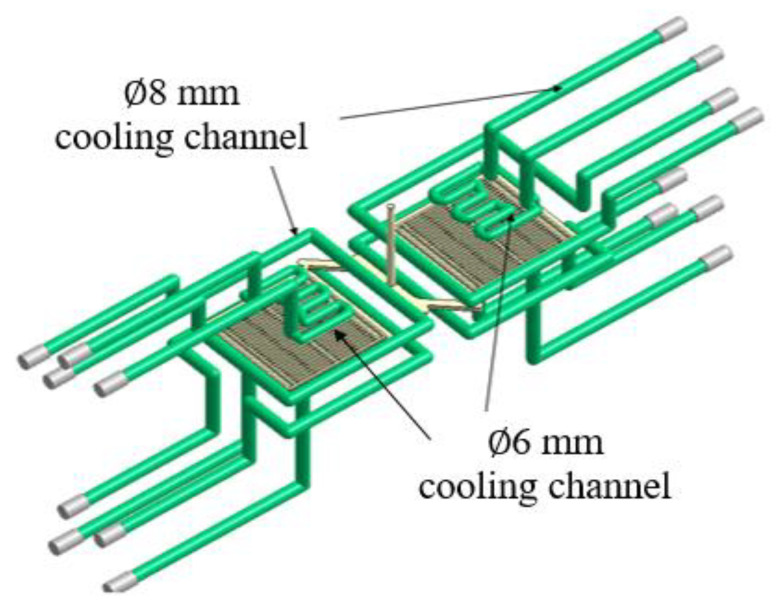
Layout of the cooling channel in the IC tray mold.

**Figure 9:**
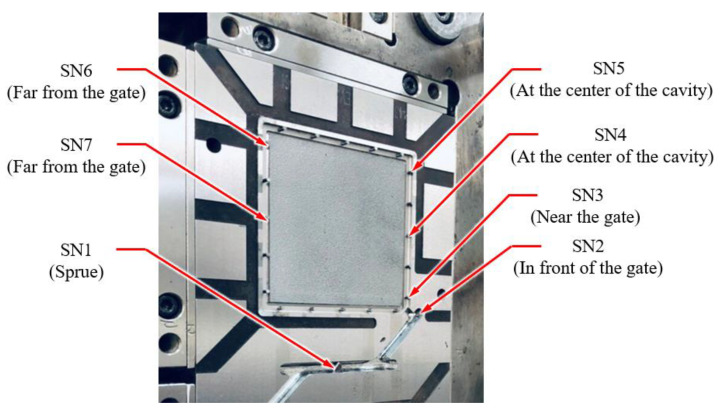
Positions of the pressure sensors installed in the cavity.

To produce IC trays, this study used an all-electric-driven injection molding machine with a clamping force of 100 tons (CT-100, Fu-Chun Shin Corporation, Tainan, Taiwan). The polymer material used was acrylonitrile–butadiene–styrene (PA-756 Chi-Mei Corporation, Tainan, Taiwan). The ratio of the flow distance to the average tray thickness was 124. Moreover, the length, width and thickness of the tray were 76, 76 and 4.4 mm, respectively. Figure 8 depicts a two-cavity injection mold used for manufacturing an IC tray with a cooling channel layout. In particular, the cooling channels at the male and female molds of each cavity were independent, which allowed the precise control of the mold temperature at each shot. Precise control of the mold temperature helps to obtain an accurate and stable geometry for the injection-molded part [32]. For sensing the cavity pressure signals, two types of pressure sensors (Futaba Corporation, Mobara, Japan) were mounted at the back of the ejector. Table 2 lists the specifications of the pressure sensors. Seven sensors were mounted in the mold. Figure 9 displays the locations of each sensor. To study the overall flow state of the melt and its response to the quality indices, seven pressure sensors were installed in the mold—one at the sprue, one in front of the gate, one near the gate, two at the center of the cavity and two far from the gate. This study assumes that the polymer melt advances in laminar flow, because the flowing behavior of the polymer melt is fountain flow and because the ratio of the flow direction distance to the average thickness is high (124 in this case), the pressure change along the thickness direction is ignored.

The performed experiment was a two-factor full-factor experiment. Table 3 lists the process parameters of the injection-molded IC tray. The injection speed ranged from 40 to 120 mm/s and the first-stage holding pressure varied from 50 to 100 MPa. At each shot, a system pressure curve and seven cavity pressure curves (SN1–SN7) were recorded. The system pressure curve was used to obtain two quality indices, namely *Ph_index_* and *PI_index_*. The seven cavity pressure curves (SN1–SN7) were used to obtain *Pp_index_* and four cavity pressure curves (SN4–SN7) for sensors installed far from the gate were used to obtain *Pr_index_*. The total number of sub experiments was 445 and each sub experiment comprised 11 quality indices. These quality indices were candidates for the input data of the MLP model and were further evaluated using the PCC algorithm. The output data were the three widths (*W1*, *W2* and *W3*) of the IC tray geometry.

### 3.2. MLP Model

**Figure 10:**
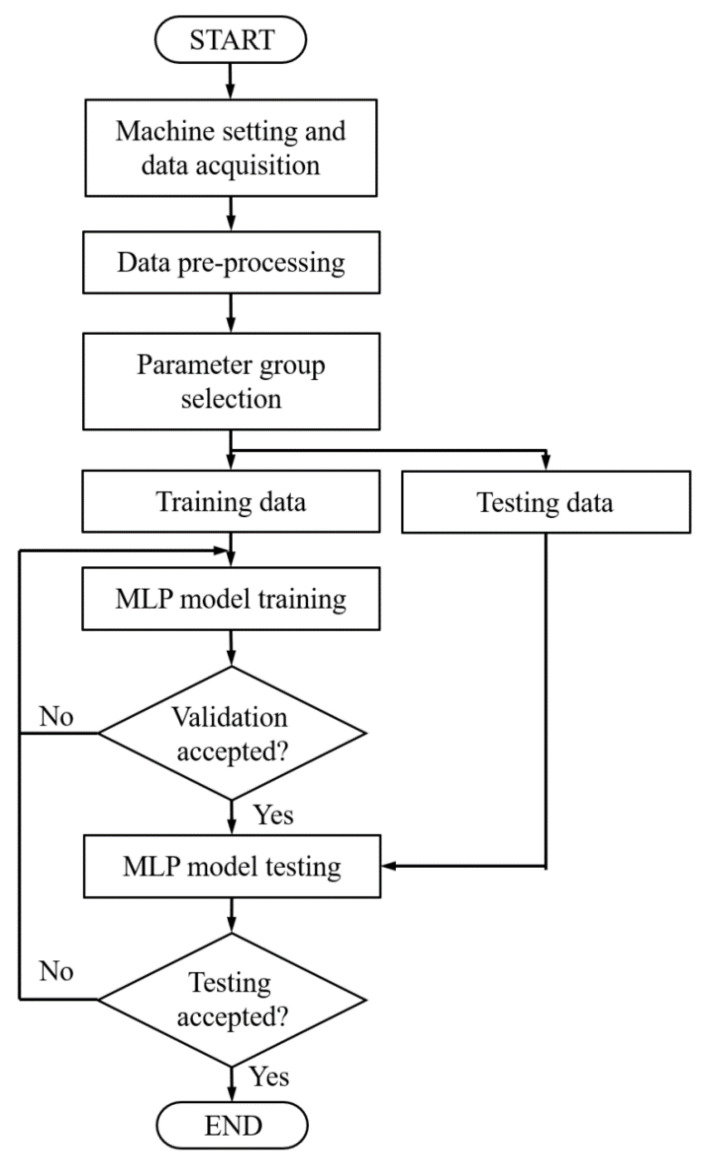
Flowchart of MLP modeling.

Figure 10 depicts the flowchart of the MLP modeling process used in this study, which begins with the preprocessing of the input data, that is, the normalization of the range of the input data between 0 and 1 by using Equation (8). A specific parameter group is then selected as the input data and the normalized data are divided into two groups—a group of 356 data points for model training and a group of 89 data points for model testing. Table 4 presents the hyperparameter design of the experimental MLP. The internal settings of the MLP are called hyperparameters, which indicate the feature settings of the training model of a group [29], including the number of iterations (epoch), batch size, number of hidden layers, number of neurons per layer and learning rate. In this study, the epoch, batch size and learning rate was set as 5000, 10 and 0.1, respectively.

**Figure 11:**
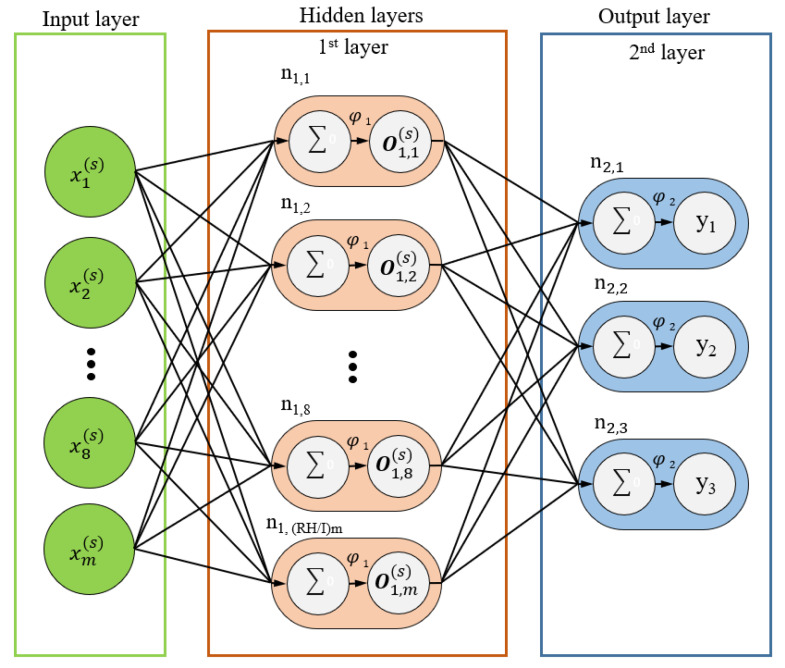
MLP framework used in this study.

Figure 11 depicts the neural network architecture used in the experiment. Practically, the number of hidden layers is usually determined by the number of quality intervals. This research aimed to distinguish quality in terms of “pass” and “fail” (i.e., “Go” and “No Go,” respectively); therefore, the model design comprised only one hidden layer. In addition, the number of nodes in each hidden layer was adjusted as multiples of the number of nodes in the input layer. The parameter $R_{H/I}$ (Equation (14)) is the ratio between the number of hidden layer nodes (*N_hidden*) and the number of input layer nodes (*N_input*). The values of *R_H/I_* were set as 1, 2 and 3 to determine the value that provided the optimal training accuracy.

(14) $$R_{H/I}=\frac{N_{hidden}}{N_{input}}.$$

In this study, the number of input nodes was related to the number of quality indices and the number of sensors embedded in the mold. The number of quality indices represents the number of injection molding process parameters that affect the quality of molded parts and the validity of the quality indices largely reflects the quality of model learning. Therefore, to perform model training with sufficient information, a large number of quality indices should be used. The number of sensors embedded in the mold is related to the sensing location, which reveals the best information regarding the injection molding process parameters. Therefore, this study evaluated the appropriate sensing location to minimize the hardware cost. In general, a balance must be achieved between the quality and cost of model training, that is, between the number of in-mold sensors and the required quality indices. This balance was examined in the present study.

### 3.3. Correlation Analysis

**Figure 12:**
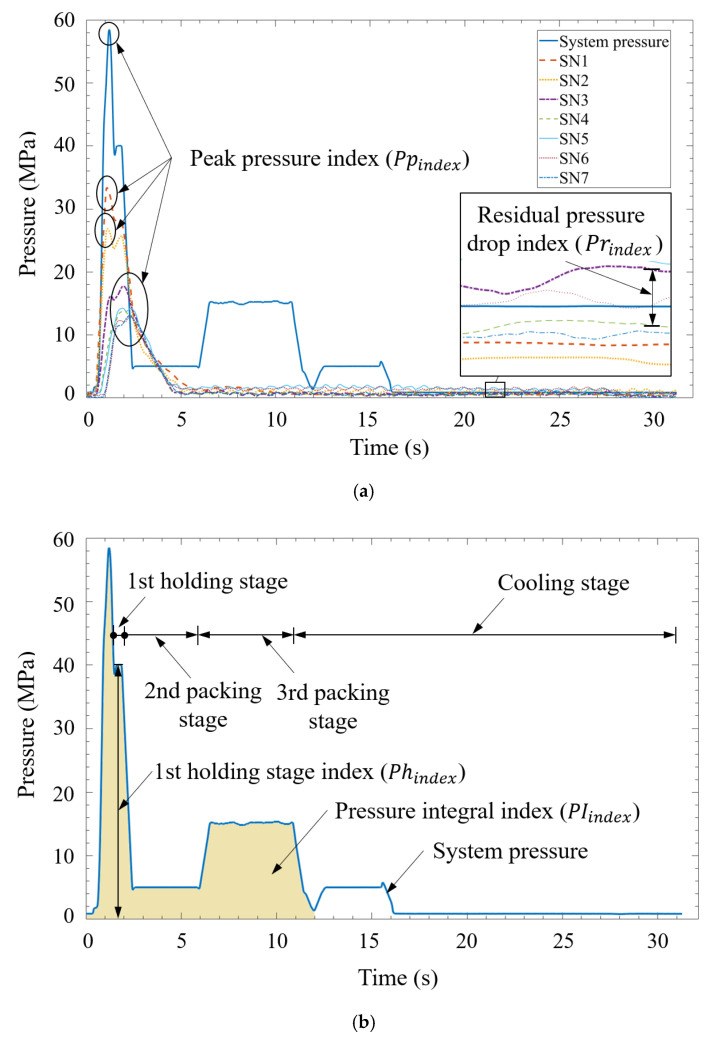
Quality indices: (**a**) peak pressure index and residual pressure drop index and (**b**) pressure integral index and first-stage holding pressure index.

Figure 12a illustrates the physical meaning of the peak pressure index ($Pp_{index}$) and residual pressure drop index ($Pr_{index}$) in the cavity pressure profiles measured by sensors 1–7. The system pressure provided the driving force for the polymer melt to overcome the resistance during mold filling and compression; thus, the polymer melt flowing through the pressure sensors had different pressures according to the flow distance. The parameter $Pr_{index}$ represents the average pressure drop from sensors 4–7 to sensor 3 in the cooling stage. This pressure drop indicated local shrinkage. Figure 12b depicts the physical meaning of the first-stage holding pressure index ($Ph_{index}$) and system pressure integral index ($PI_{index}$). The parameter $PI_{index}$ represents the momentum required for mold filling and compression, which indicated the quality of the injection-molded parts. The parameter $Ph_{index}$ represents the main packing capacity in the holding stage, which was related to the molding weight and part geometry.

Table 5 presents the PCCs of various quality indices with the width. The PCCs of $Ph_{index}$ with *W1*, *W2* and *W3* were 0.96, 0.96 and 0.97, respectively. These high correlation coefficients indicated that $Ph_{index}$ had a significant influence on the width quality. Similarly, the PCCs of $Pp_{index}$ with *W1*, *W2* and *W3* were 0.94, 0.95 and 0.94, respectively, which indicated that the driving force provided by the system pressure had considerable influence on the quality of the parts. In particular, the greater the driving force, the higher was the amount of polymer melt that could be fed into the mold cavity and the smaller was the shrinkage rate. Thus, the geometric accuracy of the molded part increased. The PCCs of $PI_{index}$ with *W1*, *W2* and *W3* were 0.79, 0.81 and 0.78, respectively, which indicated that $PI_{index}$ also had a strong correlation with the part width.

**Figure 13:**
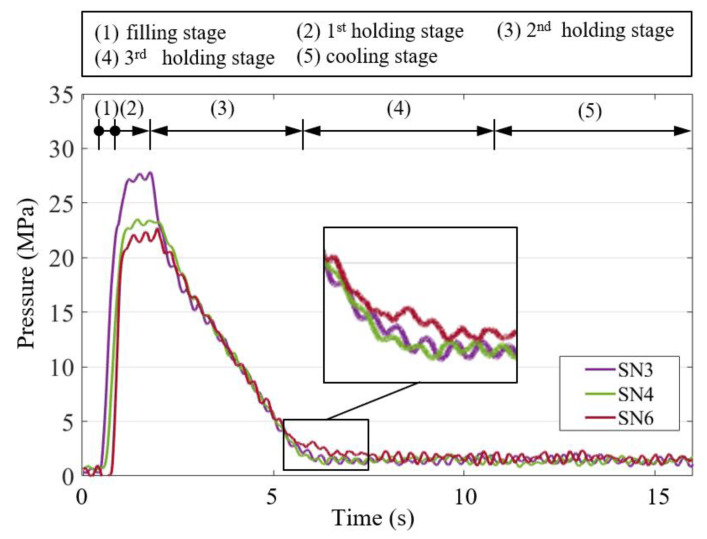
Back-flow phenomenon caused by relatively low cavity pressure distribution at the center of the cavity (SN4) during holding.

The parameter $Pr_{index}$ represents the average pressure drop between sensors 4–7 and sensor three during the cooling stage. The correlation coefficients of the three widths with the pressure drops at sensors 4 and 5 at the center of the cavity were low (approximately 0.5). However, far from the gate, the correlation coefficients of the three widths with the pressure drops at sensors 6 and 7 were high (approximately 0.95), which indicated that the residual pressure far from the gate influenced the geometric width of the molded part. This phenomenon is illustrated in Figure 13. During the holding stage, particularly in the second holding stage, the relatively low pressure distribution at the center of the cavity (SN4) caused the back-flow of the polymer melt. This unstable flow behavior resulted in a weak correlation between the pressure drop and the part quality. In this study, quality indices having high correlation coefficients (i.e., above 0.75) with the part quality were used as the input data of the MLP model.

### 3.4. Correlation Coefficients among Quality Indices

Table 6 presents the correlation among the quality indices. High correlation coefficients indicate the dependence of the indices and all the relationships among $Ph_{index}$, $Pp_{index}$ and $Pr_{index}$ were highly dependent. However, the correlation with $PI_{index}$ was relatively small (approximately 0.7). The indices $Ph_{index}$, $Pp_{index}$ and $Pr_{index}$ were related to the driving force for overcoming the flow resistance and thus had a strong correlation with it. In contrast to the aforementioned three indices, $PI_{index}$ was related to the total momentum required during mold filling and holding; thus, this index provided more information than the other three indices regarding the part quality.

This study investigated the effect of the number of quality indices on the learning of the MLP model. Table 7 lists the four groups of quality indices used in this study. Group A comprised all types of indices extracted from all the sensors. Group B comprised all types of indices from certain sensors—$Ph_{index}$ at the system pressure sensor; $PI_{index}$ at sensor 6; and $Pp_{index}$ at sensors 3, 4 and 6. The latter indicated that the indices had relatively high correlation coefficients at the sensors near the gate, at the center of the cavity and far from the gate. Similar to group B, group C comprised $Pp_{index}$ at sensor 3 (installed near the gate), which had the highest correlation coefficient with the peak pressure. Groups D1–D4 corresponded to a single type of quality index and were used to assess the feasibility of using a single quality index in model learning and quality prediction.

## 4. Results and Discussion

### 4.1. Training Accuracy of the MLP Model (R_H/I_ = 1) for Various Input Groups

**Figure 14:**
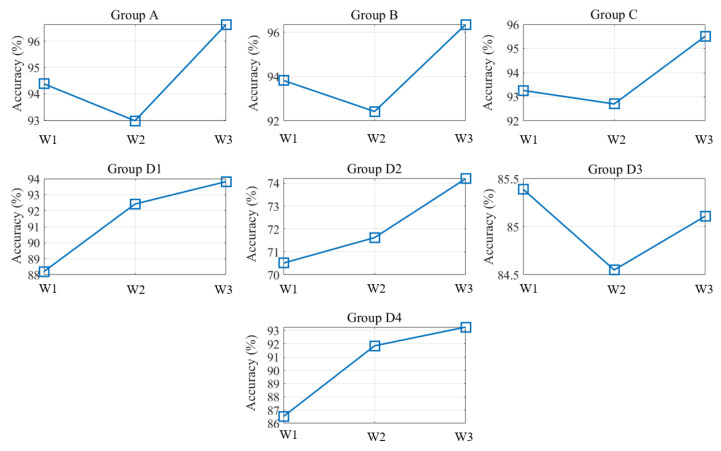
Training accuracy of the MLP model when *R_H/I_* = 1.

Figure 14 illustrates the results obtained when using an MLP model with *R_H/I_* = 1 to train the quality of *W1*, *W2* and *W3* with different groups of input data. The groups A, B and C, which represent the input numbers 11, 6 and 4, respectively, had high training accuracy (all above 92%). The training accuracy of group C was 93%, which was higher than that of groups A and B. Thus, group C required less input data than groups A and B did. The results revealed that the consideration of quality indices that represented similar physical meanings was not required. The consideration of similar quality indices did not improve the prediction accuracy but required the use of additional sensors, which increased the cost. By contrast, groups D1–D4, which used a single quality index (i.e., first-stage holding pressure index, pressure integral index, residual pressure drop, and peak pressure, respectively), had relatively poor training accuracy. Among these groups, the training accuracy of the group D2 (only 70%) was the worst, with the correlation coefficients of $PI_{index}$ with *W1*, *W2* and *W3* being 0.8. Although the correlations of $PI_{index}$ with the widths were strong, they did not meet the requirements of high-accuracy training in the MLP model. The $PI_{index}$ value extracted from the system pressure curve represents the total momentum acting on the polymer melt for overcoming the resistance of the sprue, runners, gates and cavity. The information contained in this quality index reflects all changes in the mechanisms through which the polymer melt flows; thus, the aforementioned index reflects redundant information regarding the quality of the width. The groups D1, D3 and D4 had high correlation coefficients (more than 0.9) with the widths and relatively high training accuracy (approximately 88%). Obviously, those D1 and D4, which only require a single sensor, seem to be potential in practical applications involving hardware cost. However, in this study, comprehensive and high-quality training (above 90% prediction accuracy) could not be achieved using only one set of index for model training.

As depicted in Figure 14, *W3* (95%) had the highest training accuracy for groups A, B and C, followed by *W1* (93%) and *W2* (92%). This result is consistent with Figure 13, in which *W3* far from the gate is well packed and low back-flow occurs, which introduces a consistent geometric quality and enables high-quality training of the MLP model. Similarly, *W1* near the gate should be fully packed. However, overpacking of the polymer melt may occur near the gate, which often results in inconsistent geometry and affects the training accuracy of the MLP model. With regard to *W2* at the center of the cavity, the molding quality is considerably affected by the back-flow of the polymer melt during the holding stage, which leads to inconsistent geometric shapes and relatively low training accuracy (92%).

### 4.2. Comparison of Training Accuracy when R_H/I_ = 1, 2 and 3

**Figure 15:**
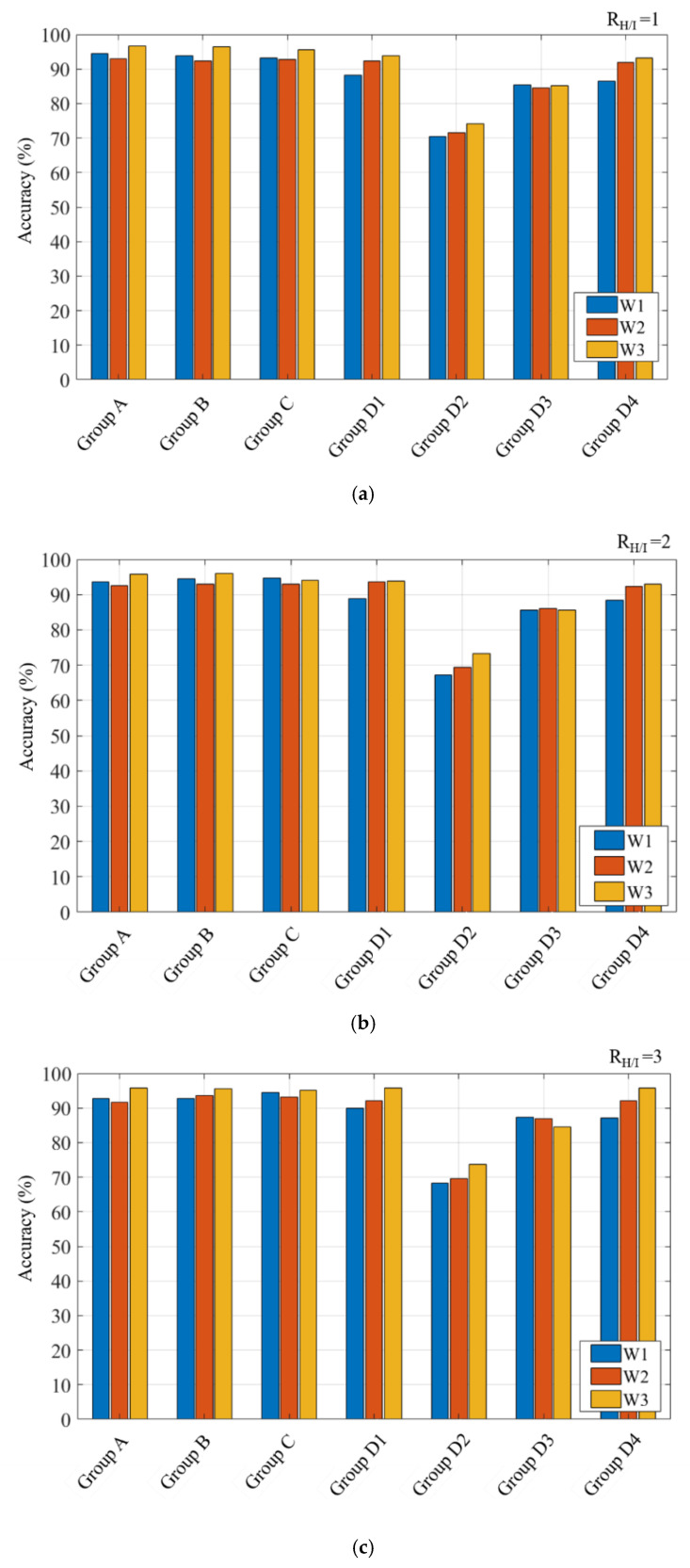
Comparison of the training accuracy when: (**a**) *R_H/I_* = 1, (**b**) *R_H/I_* = 2, and (**c**) *R_H/I_* = 3.

Figure 15 displays the results of MLP model training for various *R_H/I_* values. In this study, an increase in the number of neurons in the hidden layer did not significantly improve the training accuracy. An *R_H/I_* value of 1 is sufficient for producing high-quality training results; therefore, *R_H/I_* was set as 1 in this study.

### 4.3. MLP (R_H/I_ = 1) Prediction Accuracy for Various Input Groups

**Figure 16:**
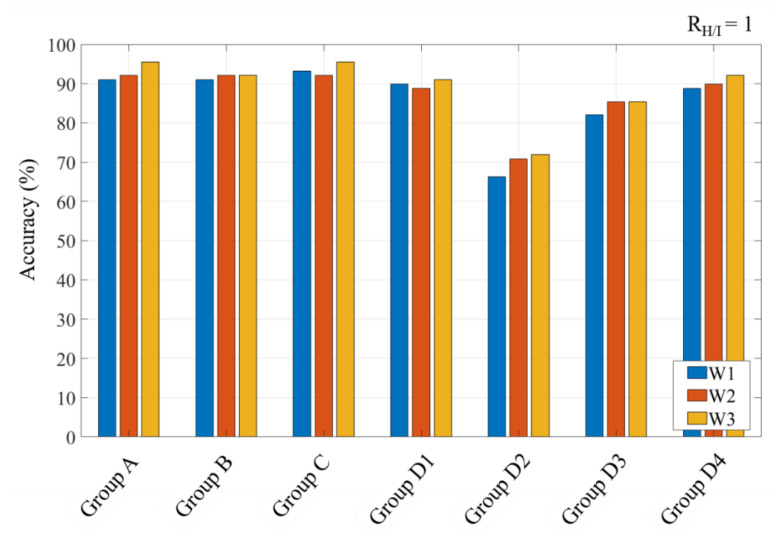
Comparison of the prediction accuracy when *R_H/I_* = 1 for various input groups.

Figure 16 depicts the prediction accuracy results obtained when using the MLP model with *R_H/I_ =* 1. The prediction accuracy rates of *W1*, *W2* and *W3* with groups A, B and C as the input data reached more than 90%, which indicated that the quality prediction performance was stable. In particular, the prediction for *W3* reached 93%. Regarding the performance of the groups D1–D4, only the prediction accuracy rates of the groups D1 and D4 were more than 90%. In general, group C exhibited the best performance in terms of prediction accuracy and cost reduction.

## 5. Conclusions

In this study, an efficient and accurate quality inspection system was developed using AI techniques to ensure that the requirements of the finished product were met, which is essential in injection molding. First, this study examined the pressure curve of the polymer melt, which reflected the flow behavior inside the mold. By referring to pressure signals, we selected four quality indices, namely the first-stage holding pressure index $\left(Ph_{index}\right)$, pressure integral index ($PI_{index}$), residual pressure drop index ($Pr_{index}$) and peak pressure index ($Pp_{index}$). We examined the correlations of these indices with the geometric width of injection-molded parts. Then, a single-layer MLP neural network model was used for quality predictions. In this model, quality indices having a strong correlation with the part quality were used as the input data. The Python module in the free software Google Colab was used to develop the MLP neural network. Then, the effect of the neuron ratio between the hidden and input layers on the training accuracy was evaluated. The results of this study can be summarized as follows:

(1) The parameters $Ph_{index}$ and $Pp_{index}$ had the highest correlation coefficients with the widths (the correlation coefficients exceeded 0.93). However, the correlation coefficients of $Pr_{index}$ were sensitive to the sensing position. The correlation coefficients of $Pr_{index}$ was high (0.92) far from the gate but very low (approximately 0.5) at the center of the cavity. Unstable flow behavior was noted at the center of the cavity. This unstable flow interfered with the back-flow of the polymer melt, which resulted in a weak correlation between the pressure drop and the part quality.
(2) All relationships among $Ph_{index}$, $Pp_{index}$ and $Pr_{index}$ were highly dependent. However, the correlation with $PI_{index}$ was relatively small (approximately 0.7). The indices $Ph_{index}$, $Pp_{index}$ and $Pr_{index}$ were related to the driving force for overcoming the flow resistance and thus had a strong correlation with it. In contrast to the aforementioned three indices, $PI_{index}$ was related to the total momentum acting on the polymer melt for overcoming the resistance of the sprue, runners, gates and cavity. The information contained in this quality index reflected all the changes in the mechanisms through which the polymer melt flowed; thus, the aforementioned quality index reflected redundant information regarding the quality of the width.
(3) The numbers of training and testing data points in the case study were merely 356 and 89, respectively. When using the MLP model at *R_H/I_ =* 1, the predictions of *W1*, *W2* and *W3* with the groups A, B and C as the input data reached more than 90%, which indicated that the quality prediction performance was stable. In particular, the prediction for *W3* reached 93%. An increase in the number of neurons in the hidden layer did not significantly improve the training accuracy in this study, that is, an *R_H/I_* value of 1 was sufficient to obtain high-quality training results.
(4) Regarding the performance of the groups D1–D4, the predictions of the groups D1, D3 and D4 had relatively high training accuracy (more than 88%). However, comprehensive and high-quality training could not be achieved using only one set of indices for model training.
(5) This study also evaluated the appropriate sensing location for minimizing the hardware cost. Theoretically, to conduct model training with sufficient information, a large number of quality indices should be used. Nonetheless, group C that exhibited the best performance in terms of prediction accuracy and cost reduction was suggested by this study.

## Figures and Tables

**Table 1:** Pearson’s correlation coefficients (PCCs) related to the correlation strength.

Range of |*r*|	Correlation Strength
0	No
0–0.25	Negligible
0.25–0.5	Poor
0.5–0.75	Moderate
0.75–1	Strong
1	Perfect

**Table 2:** Specification of the pressure sensors.

Item	Type 1: (SSB01KN08X06H)	Type 2: (SSB04KN10X08H)
Rated capacity	1 kN	4 kN
Measurement range	0.2–1 kN	1–4 kN
Stroke amount	0.02 mm	
Allowable overload	1.5 kN	6 kN
Nonlinearity	±2.0% F.S.	
Temp. limit	200 °C	
Sensitivity fluctuation	−0.03% F.S./°C max	

**Table 3:** Parameters of the injection molding process.

Item			Unit	Parameters
Melt temperature		°C		205
Mold temperature		°C		60
Backpressure		MPa		4.5
Clamping force		Tons		70
Decompression on stroke		mm		10
Holding speed limit		mm/s		80
V/P switchover position		mm		12.45
Cooling time		s		16
Holding pressure	1st stage	MPa		50, 60, 70, 80, 90, 100
	2nd stage	MPa		5
	3rd stage	MPa		15
Holding time	1st stage	s		1
	2nd stage	s		4
	3rd stage	s		5
Injection speed		mm/s		40, 50, 60, 70, 80, 90, 100, 110, 120

**Table 4:** Hyperparameters of the MLP model.

Item		Parameter
Software and version		Python 3.6.9
Integrated development environment		Google Colab
Loss function		Categorical Crossentropy
Optimizer		Stochastic Gradient Descent
Learning rate		0.1
Activation function		Sigmoid function, Softmax function
Metrics		Accuracy
Batch size		10
Epoch		5000
No. training dataset		356
No. testing dataset		89
No. neural node of	Input layer	Group A, Group B, Group C and Group D1-D4
	1st hidden layer	1*R_H/I_*, 4*R_H/I_*, 7*R_H/I_* and 11*R_H/I_*, *R_H/I_* = 1,2 and 3
	Output layer	3

**Table 5:** PCCs between the geometric widths and quality indices.

Quality Index	Sensor Position	Symbols	Pearson’s Correlation Coefficient		
			Width 1	Width 2	Width 3
$Ph_{index}$	System pressure		0.96	0.96	0.97
$PI_{index}$	System pressure		0.79	0.81	0.78
$Pr_{index}$	At the center of the cavity	SN4	0.56	0.56	0.55
	At the center of the cavity	SN5	−0.51	−0.54	−0.54
	Far from the gate	SN6	0.93	0.93	0.92
	Far from the gate	SN7	0.94	0.93	0.93
$Pp_{index}$	Sprue	SN1	0.92	0.95	0.92
	In front of the gate	SN2	0.94	0.95	0.94
	Near the gate	SN3	0.95	0.96	0.96
	At the center of the cavity	SN4	0.95	0.95	0.96
	At the center of the cavity	SN5	0.94	0.95	0.95
	Far from the gate	SN6	0.94	0.95	0.95
	Far from the gate	SN7	0.94	0.95	0.96

**Table 6:** PCCs among the quality indices.

		$Ph_{index}$	$Pp_{index}$							$PI_{index}$	$Pr_{index}$	
			**SN1**	**SN2**	**SN3**	**SN4**	**SN5**	**SN6**	**SN7**		**SN6**	**SN7**
$Ph_{index}$		1	0.94	0.98	0.99	0.99	0.99	0.99	0.99	0.77	0.94	0.97
$Pp_{index}$	SN1	0.94	1	0.98	0.96	0.94	0.95	0.95	0.95	0.73	0.93	0.94
	SN2	0.98	0.98	1	0.99	0.98	0.98	0.98	0.98	0.72	0.95	0.96
	SN3	0.99	0.96	0.99	1	0.99	0.99	0.99	0.99	0.74	0.93	0.96
	SN4	0.99	0.94	0.98	0.99	1	0.99	0.99	0.99	0.71	0.92	0.95
	SN5	0.99	0.95	0.98	0.99	0.99	1	0.99	0.99	0.71	0.93	0.96
	SN6	0.99	0.95	0.98	0.99	0.99	0.99	1	0.99	0.71	0.93	0.96
	SN7	0.99	0.95	0.98	0.99	0.99	0.99	0.99	1	0.72	0.93	0.96
$PI_{index}$		0.77	0.73	0.72	0.74	0.71	0.71	0.71	0.72	1	0.75	0.76
$Pr_{index}$	SN6	0.94	0.93	0.95	0.93	0.92	0.93	0.93	0.93	0.75	1	0.95
	SN7	0.97	0.94	0.96	0.96	0.95	0.96	0.96	0.96	0.76	0.95	1

**Table 7:** List of sensors used in each group.

Index	Sensor Position		Group						
			A	B	C	D1	D2	D3	D4
$Ph_{index}$	System pressure		●	●	●	●			
$PI_{index}$	System pressure		●	●	●		●		
$Pr_{index}$	Far from the gate	SN6	●	●	●				
	Far from the gate	SN7	●					●	
$Pp_{index}$	Sprue	SN1	●						
	In front of the gate	SN2	●						
	Near the gate	SN3	●	●	●				●
	At the center of the cavity	SN4	●	●					
	At the center of the cavity	SN5	●						
	Far from the gate	SN6	●	●					
	Far from the gate	SN7	●						
